# Green synthesis and application of GO nanoparticles to augment growth parameters and yield in mungbean (*Vigna radiata* L.)

**DOI:** 10.3389/fpls.2022.1040037

**Published:** 2022-11-10

**Authors:** Faisal Shafiq Mirza, Zill-e-Huma Aftab, Muhammad Danish Ali, Arusa Aftab, Tehmina Anjum, Hamza Rafiq, Guihua Li

**Affiliations:** ^1^ Guangdong Key Laboratory for New Technology Research of Vegetables/Vegetable Research Institute, Guangdong Academy of Agricultural Sciences, Guangzhou, China; ^2^ Department of Plant Pathology, Faculty of Agricultural Sciences, University of the Punjab, Lahore, Pakistan; ^3^ Department of Physics, University of the Punjab, Lahore, Pakistan; ^4^ Department of Botany, Lahore College for Women University, Lahore, Pakistan

**Keywords:** graphene oxide, nanoparticles, physicochemical properties, nutrient uptake, *Vigna radiata*

## Abstract

Plant growth promotion has long been a challenge for growers all over the world. In this work, we devised a green nanomaterial-assisted approach to boost plant growth. It has been reported that carbon nanomaterials are toxic to plants because they can inhibit the uptake of nutrients if employed in higher concentrations, however this study shows that graphene oxide (GO) can be used as a regulator tool to improve plant growth and stability. Graphene oxide in different concentrations was added to the soil of mungbean. It is proved that when a suitable amount of graphene oxide was applied, it had a good influence on plant growth by enhancing the length of roots and shoots, number of leaves, number of root nodules per plant, number of pods, and seeds per pod. We presume that the use of bio-fabricated graphene oxide as a strategy would make it possible to boost both plant growth and the significant increase in the number of seeds produced by each plant.

## Introduction

Mungbean [*Vigna radiata* (L.) R. Wilczek var. radiata] is a short-duration grain legume grown on 7 million hectares primarily in Asia but rapidly spreading to other parts of the world. Its seed has a protein content of 24% and is rich in antioxidants, fibre, and phytonutrients (Nair et al., 2019). It is the major summer pulse of Pakistan and is grown on 88% of the land in Punjab province, which produces 85% of the country's total output (Ullah et al., 2020). Abiotic and biotic constraints, inadequate crop management methods, and lack of quality seeds of better varieties available to farmers all contribute to the low yield of mungbean (Chauhan13 et al., 2010; Pratap et al., 2019). The major biotic constraints of mungbean include diseases which are yellow mosaic, anthracnose, powdery mildew, Cercospora leaf spot, dry root rot, halo blight, bacterial leaf spot, and tan spot (Nair et al., 2019). The importance of effective food cultivation and food security has increased due to the world's rapid population expansion and climatic change. As a result, alternative ways are being recommended to meet the requirement of food production and crop protection under severe environmental conditions (Lesk et al., 2016; Ramankutty et al., 2018).

Nanomaterials (NMs) are molecules with a diameter of 1–100 nm that play an important role in plant development and production, especially in stressful situations. Moreover, these nanomaterials are used as fertilizers which reduce nutrient loss while also lowering fertilizer input. The graphene oxide nanoparticles (GO NPs) are being widely used in a variety of fields, including basic science, medical and energy (Aazami et al., 2022). Studies are being done utilizing carbon nanomaterials to solve problems in agriculture such as plant disease, pesticides, and stress (Vinković et al., 2017). Although GO NPs have the ability to control plant growth and development, its mechanism is unknown. Plant reaction to GO has been linked to the reactive oxygen species (ROS) pathway, according to research (Siddiqi and Husen, 2017).

Scientists are trying to minimize the negative impacts of nanoparticles which are synthesized using chemical processes, incorporating green synthesis of nanoparticles which is found to be very effective (Gur et al., 2022). The nanomaterials synthesized through chemical methods are not ecofriendly since the chemicals utilized in the process of chemical synthesis are often toxic and flammable (Jain et al., 2009). *N. sativa* belonging to family Ranunculaceae is a medicinal plant that is used all over the world. Because of its diverse potential, it is one of the most important medicinal plants and is commonly known as black seed. The black seeds are being used as natural food additions and have medical properties such as bronchodilator, insulinotropic, anticancer, antinociceptive, anti-inflammatory, hypoglycemic, hepatoprotective, neuroprotective, antihistamine, and antiulcer properties (Ahmad et al., 2013).

Graphene-based nanomaterial applications in agriculture have received a lot of attention. Researchers are employing GO NPs in agricultural crop enhancement. Soil-based graphene oxide treatment increased the physicochemical qualities of the soil (Shekari et al., 2017). On addition, a study in *Silybum marianum* found that using graphene-oxide improved chlorophyll content and relative water content (RWC), as well as improving plant growth and yield (Safikhan et al., 2018). GO at optimum concentrations improved *Arabidopsis thaliana* L. stability and growth, as shown by increases in root length, leaf area, leaf number, and flower bud production (Park et al., 2020). Under salt stress, graphene oxide was employed to improve the growth and yield of pearl millet (*Pennisetum glaucum* L.) and it gave ideal results at 20 mg/L concentration (Mahmoud and Abdelhameed, 2021).

In this research, we showed that low concentrations of graphene oxide nanoparticles (GO NPs) enhanced good plant development and stability, implying that this nanomaterial can have beneficial effects on plant growth and quality. Our findings offer insight into the logical design of an effective nanomaterial-assisted culture system that may be utilized to speed up both plant growth and fruit ripening.

## Materials and methods

### Chemicals and reagents

The graphite (90%), hydrogen peroxide (H_2_O_2_), potassium permanganate (KMnO_4_), ferrous chloride (FeCl_2._6H_2_O), ferric chloride (FeCl_3_.4H_2_O), and sulfuric acid (H_2_SO_4_) were purchased from Sigma-Aldrich. The chemicals were analytical grade. Seeds of *Nigella Sativa* were purchased from a local market in Lahore, Pakistan. The experiment was performed at the Experimental Station, Department of Plant Pathology (31°29’42.2664” N, 74°17’49.1316” E, 217 m altitude), Faculty of Agricultural Sciences, University of the Punjab in 2021-2022. A semi-arid climate (Köppen climatic classification BSh) with an average temperature of 40°C, 350 mm of annual precipitation, and a rainy season from July to September characterizes the area.

### Preparation of *Nigella Sativa* seed extract

*Nigella Sativa* seeds (30 g) were washed, powdered, and boiled for 15 minutes in 150 mL of double-distilled water. The aqueous seed extract was then filtered through Whatman No. 1 filter paper and allowed to cool. The seed extract was kept in a sterile container at 4 °C for future use.

### Synthesis of graphene oxide

Hummer's technique was used to prepare the GO (William et al., 1958). In the standard procedure, 3 g of graphite and 3 g of NaNO_3_ were added to 150 mL of H_2_SO_4_ and the mixture was kept at 0 °C with constant stirring for 3 hours. Then, KMnO_4_ was slowly added, and the suspension was constantly stirred for 2 hours. Then, 150 mL of distilled water was gently added, and the suspension was stirred for 30 minutes after. Then, H_2_O_2_ was added cautiously to the above suspension and stirred for 20 min. Finally, the suspension was transferred to a 1L beaker and multiple washings with double distilled water were performed until the neutral pH was achieved. Then, it was filtered and dried at 70°C. This resultant mixture was (GO).

### Synthesis of graphene oxide nanoparticles

**Figure 1:**
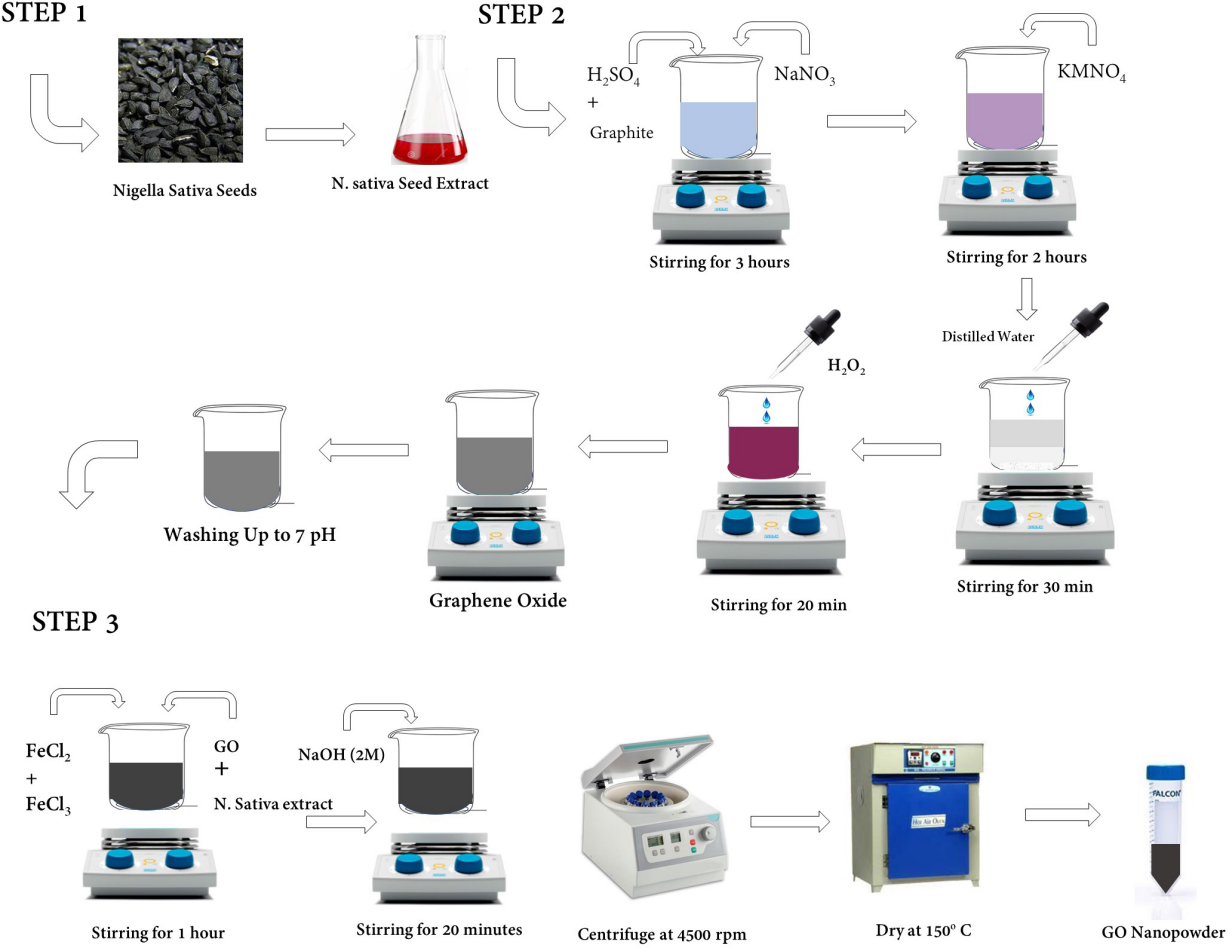
Schematic representation of the synthesis of graphene oxide nanoparticles.

A mixture of 1g of GO and 70 mL of water was sonicated for 1.5 hours. The mixture was then given a 20-minute stir after the addition of 20 mL of *N. sativa* seed extract. Then, a solution of FeCl_2_.4H_2_O (6.33 g) and FeCl_3_.6H_2_O (16.22 g) was mixed separately in 200 mL of distilled water and stirred for 30 minutes, producing a dark green colour and mixed into the solution while constant stirring. Then, sodium hydroxide NaOH (2M) was mixed into the solution and stirred for 20 minutes. The resulting mixture was put to centrifuge at 4500 rpm for 15 minutes. After centrifugation, supernatant was discarded, and the pallet was dried in a hot air oven at 150°C. The powder was crushed in a mortar and pestle to produce the desired tiny graphene oxide nanoparticles. The graphene oxide nanoparticles were then transferred to a sterile falcon tube and used for additional optical and structural characterizations. **Figure 1** depicts a schematic representation of the synthesis of graphene oxide nanoparticles.

### Characterization of green synthesized graphene oxide nanoparticles

The X-ray diffraction studies of GO NPs were performed on a Rigaku 600Miniflex X-ray diffraction (XRD) instrument with Cu k-α radiation (λ= 1.5412) in the scanning range of 100 -800. UV–visible (UV–vis) spectrum was taken in the wavelength range of 200–600 nm using Agilent Technologies Cary 60 UV–vis to confirm the absorbance of GO NPs and to observe the changes in absorbance caused by variations in reaction conditions. Fourier transform infrared (FTIR) spectra of all samples were recorded in a range of 650–4000 cm−1 in order to identify the characteristic functional groups present on the surface of the GO. Morphological characterization of the green synthesized graphene oxide nanoparticles was performed using high-resolution scanning electron microscopy (HR-SEM).

### *In vivo* application of graphene oxide nanoparticles

To create each GO NPs solution, GO NPs was disseminated in water using sonication. The soil was fumigated using and 10-12 kg soil per pot was filled in 12-inch earthen pots (having 0.264, 0.600 and 0.520 g urea, triple super phosphate and potash respectively in accordance with 40-60-40 kg N, P and K per hectare; half of the urea and other fertilizers applied before sowing and the rest was applied during the vegetative stage of the plants). The mungbean seeds were provided by the Nuclear Institute for Agriculture and Biology (NIAB), which is situated in Faisalabad, Pakistan. A mungbean variety NM-2011 was selected for pot experiment. In each pot, five holes (1cm depth) were drilled, and 3 seeds were planted in each hole. The treatments applied under *in vivo* trial are given in (**Table 1**). Every week, the pots were irrigated with 50 mL of an aqueous solution of GO NPs in accordance with the treatments listed in **Table 1**, and the growth and germination were observed. The entire experiment was conducted in triplets. Every two days, the perimeter of the mungbean plant was checked.

**Table 1:** Treatments for evaluating the efficacy of green synthesized GO NPs to augment mungbean plant growth and yield *in vivo*.

Sr	Treatments	Label
1	Control (without GO treatment)	C
2	GO NPs solution (300 mg/L)	T1
3	GO NPs solution (600 mg/L)	T2
4	GO NPs solution (900 mg/L)	T3
5	GO NPs solution (1200 mg/L)	T4
6	GO NPs solution (1500 mg/L)	T5

### Total chlorophyll and carotenoids measurements

The total Chl concentration was estimated by extracting from 0.05 g frozen leaf samples in the dark with 10 ml of 80% acetone for 24 hours (Gong et al., 2022). A spectrophotometer (UV-2550 Shimadzu, Japan) was used to measure the concentrations of Chl *a*, *b*, and total Chl (mg/L) in the supernatant. In each treatment, three plant samples were examined. Carotenoids were measured following (Alam et al., 2016), with minor adjustments. First, 2 g of dried sample was homogenized for 5 minutes in 40 mL acetone, 60 mL n-hexane, and 0.1 g MgCO_3_ after which it was vacuum filtered. The filtrate was then washed twice in 25 mL acetone and once in 25 mL n-hexane. The filtrate was put into a separatory funnel and rinsed with 100 mL of distilled water five times. A spectrophotometer was used to determine the absorbance value of 436 nm. The absorbance levels were then quantified by comparing them to the β-carotene reference curve.

### Estimation of total protein content

Total protein content of 2 g mungbean seeds from treated and non-treated plants was estimated by the following (Rizvi et al., 2022).

### Measurement of enzymatic activity and malondialdehyde content

A total of 0.5 g of fresh mungbean leaves were crushed in 3 mL of a 50 mM ice-cold phosphate buffer (pH 7.0), centrifuged at 10,000 rpm for 20 min at 4 °C, and the supernatant was kept at 4 °C until an enzymatic activity assay.

The inhibition of nitrobluetetrazolium (NBT) reduction served to measure the superoxide dismutase (SOD) activity (Giannopolitis and Ries, 1977). Enzyme extract, phosphate buffer, Na2EDTA, NBT, methionine, and riboflavin were included in the reaction mixture. For 20 minutes, the mixture was exposed to 4000 lx of illumination. The reaction was terminated in complete darkness, and the absorbance at 560 nm was measured. One SOD unit indicates the enzyme concentration that inhibits NBT photochemical degradation by 50%.Peroxidase (POD) activity was measured by the guaiacol method (Omran, 1980). Enzyme extract was mixed with a solution containing phosphate buffer, H_2_O_2_, and guaiacol in 3 mL. The increase in absorbance was monitored at 470 nm.

The depletion of H_2_O_2_ at 240 nm was used to measure the catalase (CAT) activity (Aebi, 1984). Phosphate buffer, H_2_O_2_, and the enzyme extract made up the reaction mixture. At 240 nm, the absorbance decrease was seen. The amount of enzyme needed to break down 1 µmol of H_2_O_2_ per minute is described by a CAT unit.

Thiobarbituric acid (TBA) reaction was used to measure the amount of malondialdehyde (MDA) (De Vos et al., 1991). Trichloroacetic acid (5% w/v) was used to dissolve leaf samples and centrifuged for 10 min at 4000 rpm. The supernatant was then added to 2 mL of 0.67% (w/v) TBA, which was then heated for 15 minutes in a water bath. The liquid was immediately cooled, and then centrifuged once again for 10 min at 4000 rpm. By measuring the absorbance at 532, 60, 0, and 450 nm and converting it to µmol g^-1^ FW, the MDA content was calculated.

### Nutrient analysis

After harvest, dried leaf samples (0.2 g) were processed with concentrated H_2_SO_4_ (98%) and H_2_O_2_ (30%), followed by the determination of nitrogen (N) using Nessler’s colorimetry and the measurement of phosphorus (P) using molybdenum-antimony anti-spectrophotometry (Bao, 2000). The quantities of Cu, Zn, Fe, Mn, Mo, B, Si, and K were assessed by adding 0.2 g of dry leaves to a tube containing concentrated HNO_3_ (67%) and H_2_O_2_ (30%) in a 3:1 (v/v) ratio. The samples were degraded at 120–150 °C on an electric hot plate until the solution was transparent. The samples were then digested at 120–150 °C on an electric hot plate until the solution was transparent. Deionized water was used to dilute the digest to a volume of 50 mL, and inductively coupled plasma mass spectrometry was used to quantify it (ICP-MS).

### Data recording of growth parameters

Data on growth parameters were taken by following standard practices. Harvesting was done by pulling plants out of the soil along with their roots. For later use, the samples were kept in polythene bags and labelled. The following parameters were recorded after harvesting the crop and during the growth period for each treatment.

Shoot and root length, number of leaves, number of pods, number of seeds per pod, pod size, fresh and dry weight of shoots, fresh and dry weight of roots, number of nodules per root, fresh and dry weight of pods.

### Statistical analysis

The obtained data was statistically analyzed using the DSASTAT (Onofri, Italy). For average values, a one-way ANOVA variance analysis was performed and the significant differences among the values were determined using Tukey’s Multiple Range test. For the applied variables, a significant value of P ≤ 0.05 was achieved.

## Results

### X-ray diffraction

**Figure 2:**
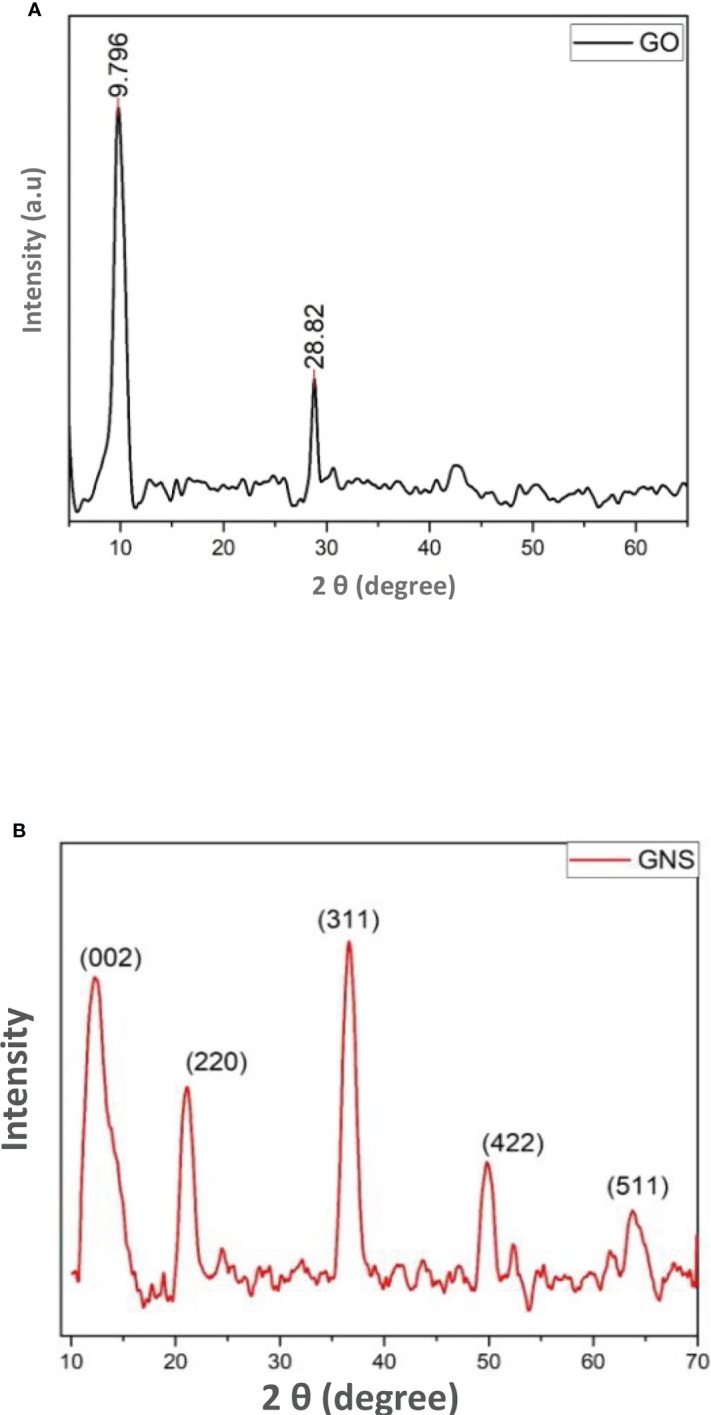
**(A)** = XRD pattern of synthesized graphene oxide; **(B)** = XRD pattern of green synthesized graphene oxide nanoparticles.

The X-ray diffractometry of the prepared sample was examined through diffractometer with wavelength of 1.5405 Å. The peaks of GO were detected at angle of diffraction 9.8 and 28.9 against planes of (001) and (002) as a result of oxygen based functional groups and graphite incomplete oxidation (**Figure 2A**). This incomplete oxidation observed due to graphite sheets that confirms that GO is formed (Ali et al., 2021). On the other hand, the extreme peak at 28.82 is detected as a result of disordered structure of carbon that is responsible of disordered assembling of graphene (Ali et al., 2022).

It was clearly obvious that the majority of oxygen-containing functional groups were eliminated throughout the reduction process. Nanocomposites exhibit intense diffraction peaks indexed to the (2 2 0), (3 1 1), (4 2 2), (5 1 1), and (4 4 0) planes which appear at 2θ = 11, 30, 35, 54, and 59 respectively, that are consistent with the standard XRD data from the JCPDS card (19-0629) to compare it with the face centered cubic (fcc) structure of Fe_3_O_4_ (**Figure 2B**) (Shen et al., 2012). The small size of magnetite nanoparticles is shown by the broadness of the diffraction peaks.

### UV-vis spectroscopy

**Figure 3:**
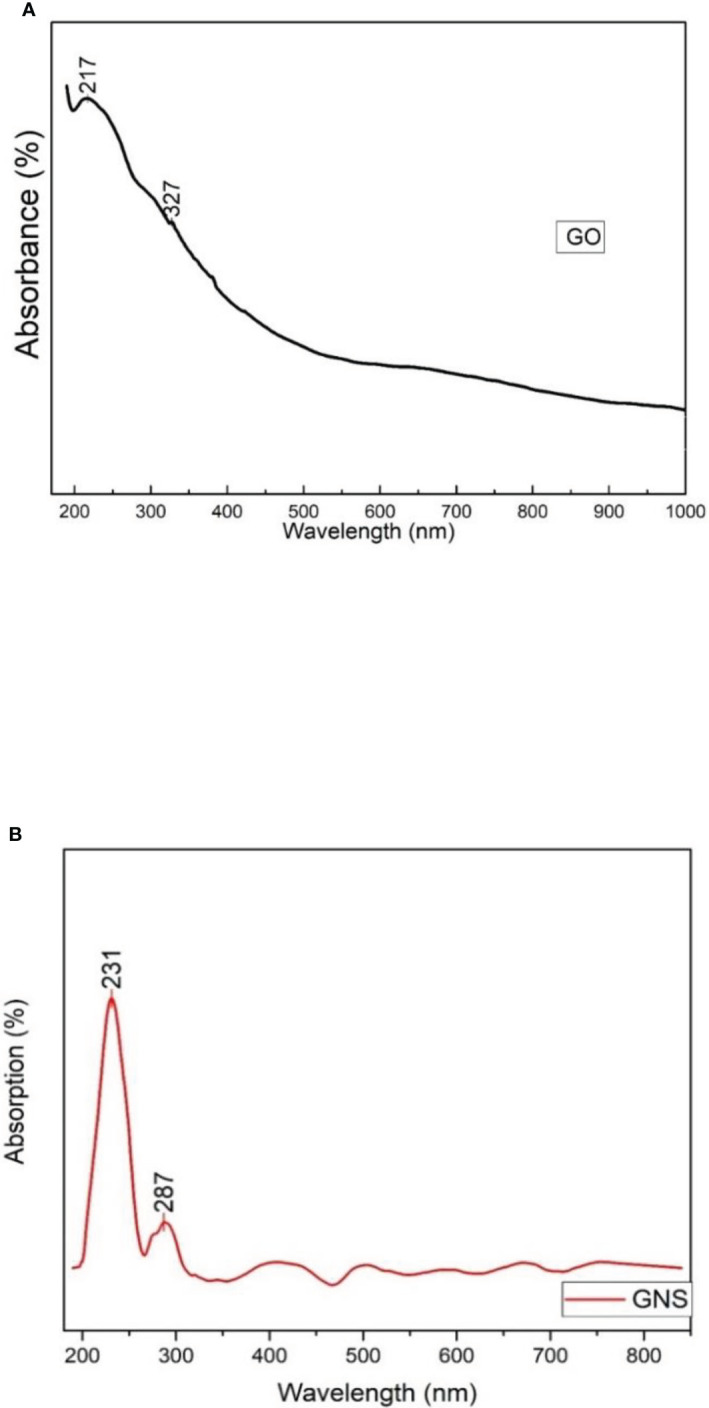
**(A)** = UV-vis analysis of synthesized graphene oxide; **(B)** = UV-vis analysis of green synthesized graphene oxide nanoparticles.

The UV absorption spectra of different Graphene oxide suspensions in DI water were observed to understand the transitions from the ground state to the excited states. Spectrum of UV-Vis. spectrum of GO was noticed at 217 and 327 nm (Ali et al., 2021, 2022). Some changes were observed during treatment of peaks and reduced values are shown in graph. It has been noticed that there are several defects observed in material due to the edge states, size and functional groups. Two peaks were observed in GO spectra. The first extreme peak is detected at 217 nm and the other peak is noticed at 327 nm (**Figure 3A**). Due to C-C bonding a peak of π to π* transition was observed at 217 nm and because of n to π* transition a shoulder peak was detected at 327 nm (Ali et al., 2021).

**Figure 3B** shows the UV-vis spectrum for GNS and exhibits a strong peak at around 231 nm and a wide shoulder peak at 287 nm. The peak noticed at 231 nm is because of the π- π * transition of C-C bonds. Whereas the peak observed at 287 nm is because of the n- π* transition and due to the presence of links that resemble epoxide (C-O-C) and peroxide (-O-O) in the structure of GNS. The absorption band of reduced GO from 231 nm to 287 nm could be because of the restoration of π electronic conjugation after reduction inside the graphene sheets. According to the absorption spectra of GNS nanocomposite, the peak at 231 nm indicates a red shift caused by a reduction in electronic conjugation of π electron, which increases the distance between HOMO and LUMO. This blue shift is also supported by the disappearance of the broad absorbance shoulder in GNS when compared to GO.

### Fourier transform infrared spectroscopy

**Figure 4:**
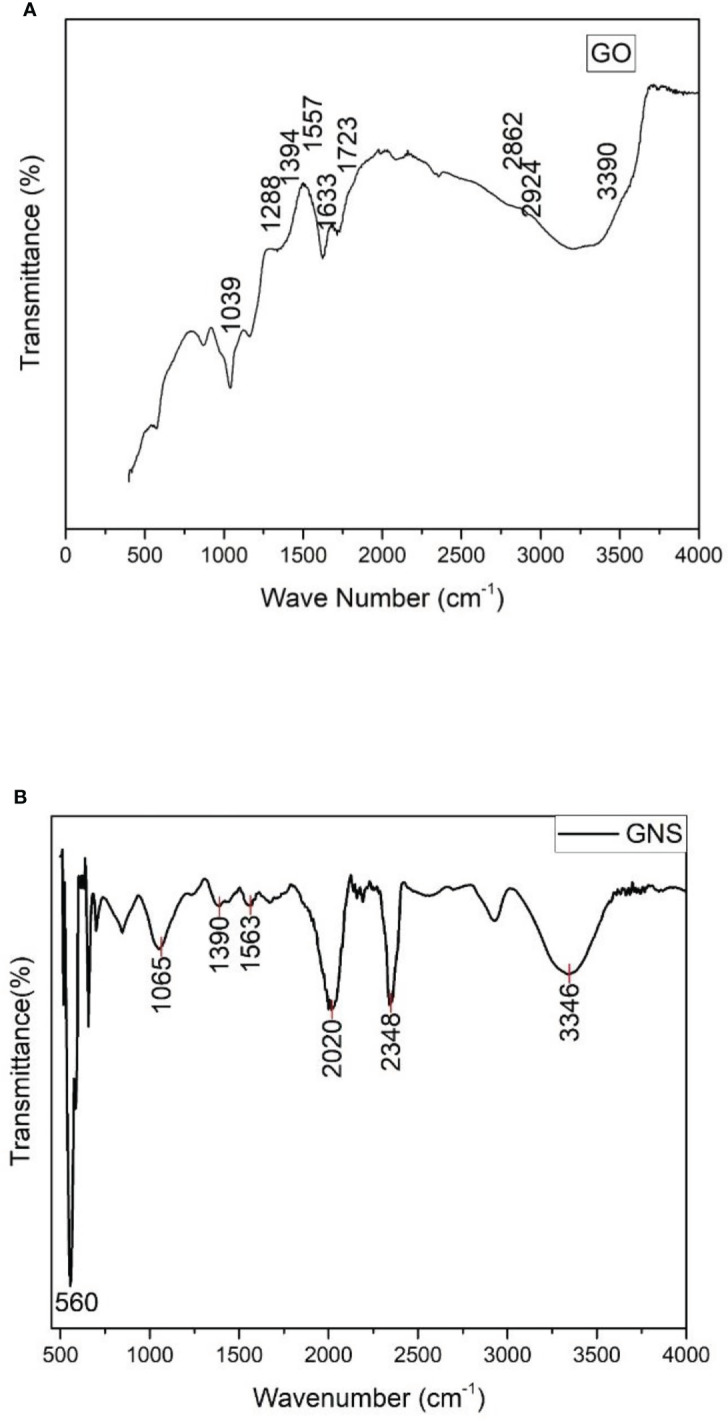
**(A)** = FTIR spectrum of synthesized graphene oxide; **(B)** = FTIR spectrum of green synthesized graphene oxide nanoparticles.

The functional groups were noticed due to the oxidation of graphite and response of these functional groups is detected in the form of peaks (**Figure 4A**). The peaks 1039, 1288, 1394, 1557, 1633, 1723, 2862, 2924, and 3390 (cm^-1^) are noticed due to the presence of alkoxy C-O vibration, epoxy, C=O deformation, O-H bond, and O-H stretching. The peaks of absorbed water were detected at 2862, 2924, and 3390 cm^-1^ due to oxidation process and these peaks are attributed to O-H stretching. The skeleton vibration of graphite or Sp^2^ vibration in graphite sheet was due to carbon and is noticed at 1633 cm^-1^. By the presence of epoxide (C-O-C) and carboxyl (COOH) groups, the extremes were observed at 1039 and 1723 cm^-1^.

According to the FTIR spectra observed, oxidized graphene contains several peaks which contain oxygen as functional group (**Figure 4B**). The spectra peaks show dissimilarity to pure graphene and C=O band expressing stretching vibration peak between 1700-2200 cm^-1^ and other oxygen bonds, i.e., C-O-C, C-O-H, and C-O, between 1546-1045 cm^-1^ (Golsheikh et al., 2013; Yan et al., 2014). The O-H bond expressing bending vibrational modes can be related to the wavenumbers 3271 cm^-1^ and 570 cm^-1^. The significantly identified peaks of 3715 cm^-1^ and 715 cm^-1^ can be predicted to be for the stretching and bending vibrations of C-H bond (Hayyan et al., 2015). At 1747 cm^-1^ (Guo et al., 2009; Marcano et al., 2010), C=C from unoxidized sp^2^ CC bonds was detected, whereas the peak at 2356cm^-1^ was brought on by CO_2_ absorption from the atmosphere (Shen et al., 2012).

### SEM analysis

**Figure 5:**
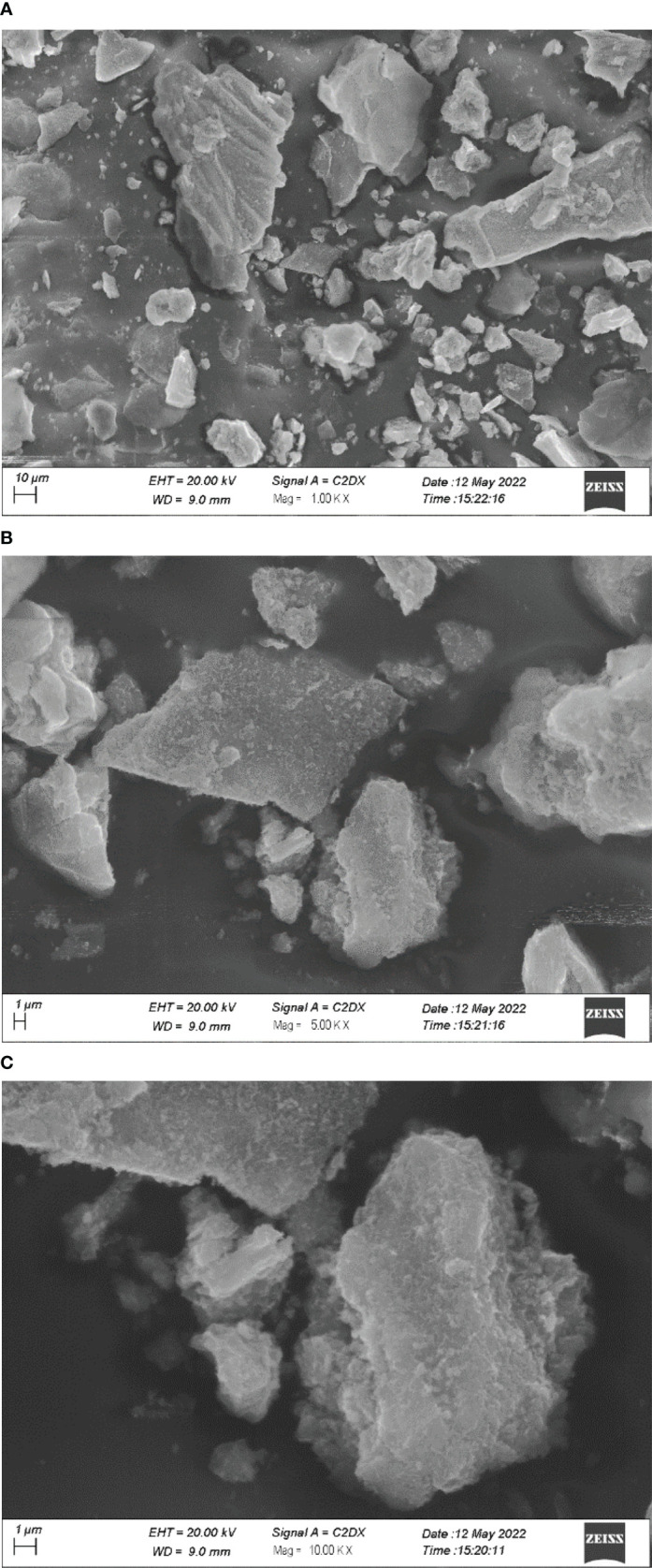
SEM images of graphene oxide nanoparticles under different magnifications **(A)** 1 KX; **(B)** 5KX; **(C)** 10KX.

**Figure 6:**
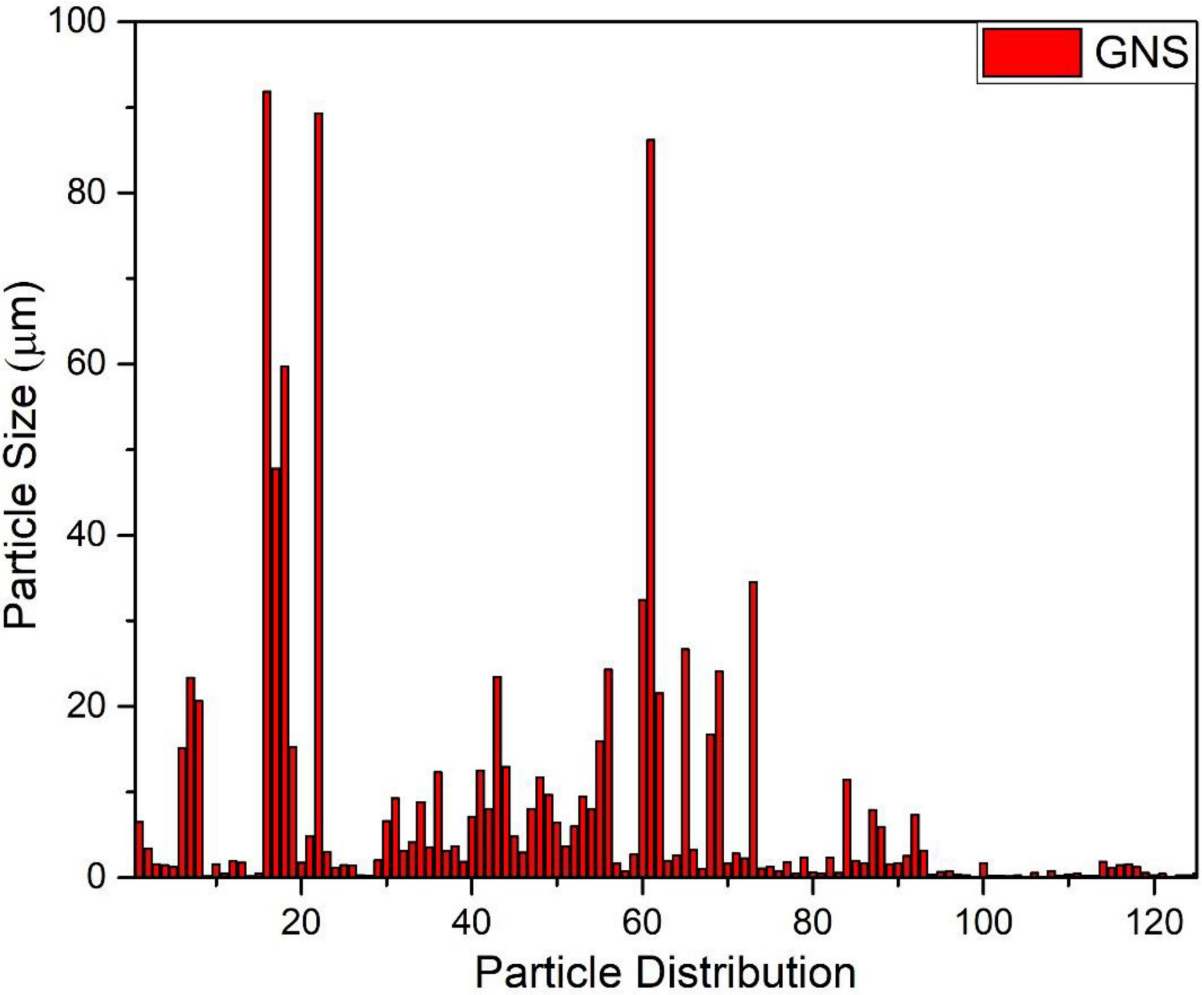
Graphical representation of particles distribution size of green synthesized graphene oxide nanoparticles.

**Figure 5** depicts SEM images of GO NPs. From the images, it can be seen that the particles are homogenously distributed and in the form of a blob/fleck shape. The particle size is in the range of 10 nm to 100 μm. The distribution graph of particles presents the size range and in-homogeneity of the particles. It is suggested that the in-homogeneity of the particles was observed due to doping of Fe^2+^ and Fe^3+^ in GO. The distribution graphs show the distribution of particles on the sheets of GO (**Figure 6**).

### Chlorophyll contents

Chlorophyll contents were assessed in order to look into how the GO NPs affected the amount of photosynthetic pigments in the mungbean plants (**Table 2**). The mungbean plants performed positively in the applied treatments of GO NPs in terms of chlorophyll contents. In the current study, the contents of chlorophyll *a*, chlorophyll *b*, and total chlorophyll gradually increased as the concentration of GO NPs increased up to 1200 mg/L. When compared to the control, the amount of chlorophyll *a* increased by 61.83%, chlorophyll *b* increased by 69.21%, and the amount of total chlorophyll increased by 64.18% at 1200 mg/L concentration. But when these plants were exposed to a higher concentration of 1500 mg/L GO NPs, their photosynthetic contents were decreased. In comparison to the control plants, chlorophyll *a*, chlorophyll *b*, and total chlorophyll of mungbean plants all decreased by 7.90%, 9.87%, and 8.53%, respectively.

**Table 2:** Effects of different concentrations of green synthesized GO NPs on physio-biochemical parameters of mungbean plants.

GO Treatments (mg/L)	Chlorophyll *a*	Chlorophyll *b*	Total chlorophyll	Carotenoids	Total proteins
**0**	0.360 ± 0.009^e^	0.168 ± 0.012^c^	0.529 ± 0.013^e^	13.699 ± 0.281^d^	2.12 ± 0.009^e^
**300**	0.407 ± 0.013^d^	0.211 ± 0.003^b^	0.619 ± 0.017^d^	15.712 ± 0.213^c^	2.25 ± 0.014^d^
**600**	0.482 ± 0.008^c^	0.225 ± 0.008^b^	0.707 ± 0.017^c^	18.775 ± 0.417^b^	2.67 ± 0.008^c^
**900**	0.538 ± 0.003^b^	0.259 ± 0.005^a^	0.798 ± 0.003^b^	20.494 ± 0.577^a^	3.47 ± 0.012^b^
**1200**	0.583 ± 0.011^a^	0.285 ± 0.008^a^	0.868 ± 0.012^a^	21.828 ± 0.333^a^	3.96 ± 0.013^a^
**1500**	0.331 ± 0.004^e^	0.152 ± 0.005^c^	0.483 ± 0.002^e^	12.463 ± 0.577^d^	1.24 ± 0.005^f^

Data represent the mean ± standard error. Different letters denote statistically significant differences between treatments as evaluated by the Tukey’s Multiple range test at the P<0.05 level. C= without GO NPs application (control; T1= 300 mg/L; T2= 600 mg/L; T3= 900 mg/L; T4= 1200 mg/L; T5= 1500 mg/L.

### Total carotenoid content

In order to determine how the GO NPs affected the amount of photosynthetic pigments in the mungbean plants, the carotenoid content was assessed (**Table 2**). Treatment of GO NPs on mungbean plants resulted in an increase in carotenoid contents. The total carotenoid levels of mungbean seedlings were observed to increase in response to GO NPs (300-1200 mg/L). The carotenoid content increased by 59.34% at 1200 mg/L in comparison to the control. However, the carotenoid content was reduced when they were subjected to a greater concentration of 1500 mg/L. In the case of 1500 mg/L concentration, the carotenoid contents of mungbean plants were reduced by 9.02% as compared to the control plants.

### Total protein content

The total protein content was determined in all the mungbean samples, and it was observed that the protein content increased with an increasing concentration of GO and showed a decline at concentration of 1500 mg/L (**Table 2**). The highest amount of protein (%) was found at a treatment of 1200 mg/L (3.96 %) and the lowest was at 1500 mg/L (1.24 %).

### Enzymatic activity

**Figure 7:**
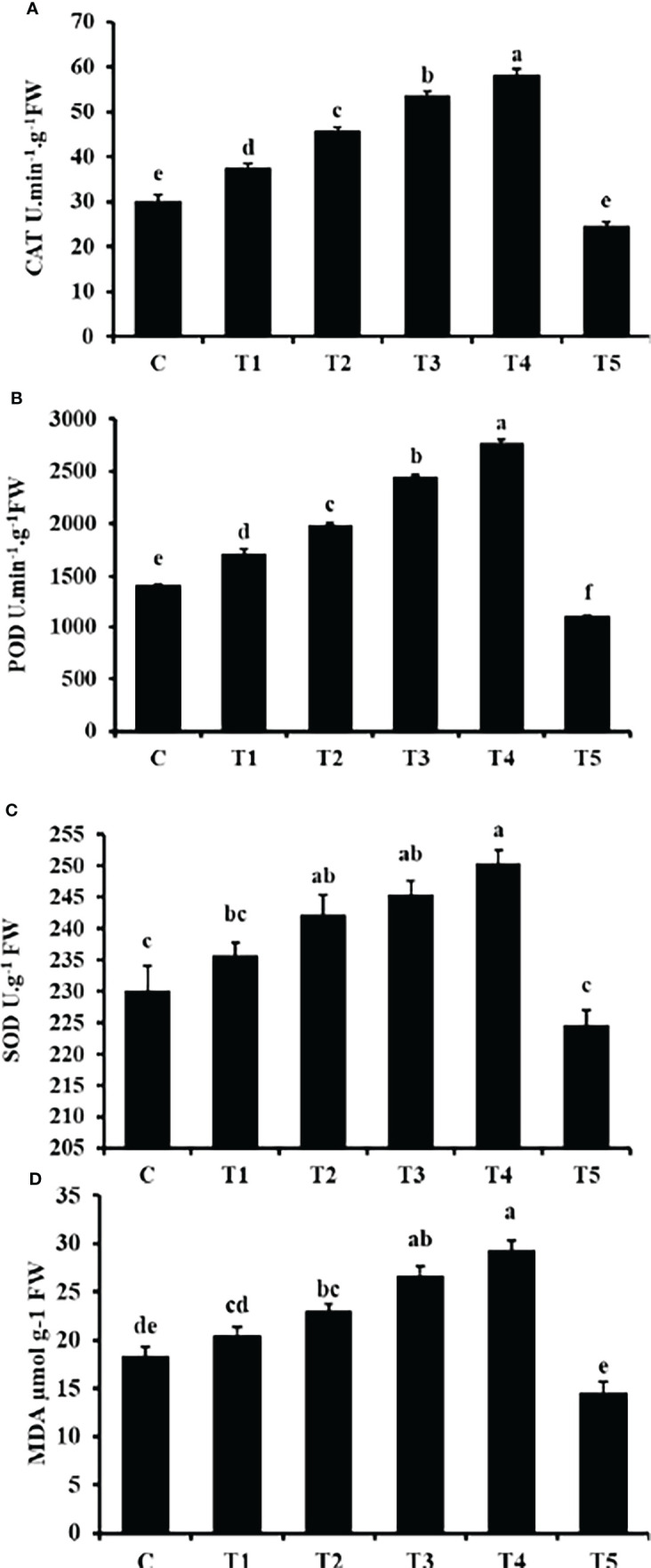
Effects of different GO NPs treatments on mungbean CAT **(A)**, POD **(B)**, SOD **(C)** activities and MDA **(D)** content. The error bars represent the standard error of means. Different letters denote statistically significant differences between treatments as evaluated by the Tukey’s Multiple range test at the P < 0.05 level. C= without GO NPs application (control); T1= 300 mg/L; T2= 600 mg/L; T3= 900 mg/L; T4= 1200 mg/L; T5= 1500 mg/L.

**Figure 7** shows that the CAT, POD and SOD activity significantly increased with increasing GO concentration except for T5 (1500 mg/L) GO. The peak values of CAT, POD and SOD activity occurred at T4 (1200 mg/L) GO, with an increase of 94.13%, 96% and 8.85%, respectively, compared with the control. While at T5 (1500 mg/L), the decreases in CAT, POD, and SOD were 17.98%, 21.84%, and 2.3%, respectively.

### Malondialdehyde content

The MDA content showed a consistent increase with increasing GO concentration except for T5 (1500 mg/L) (**Figure 7D**). Compared to the control, the MDA content in the T5 (1500 mg/L) GO treatment decreased by 20.56% and increased by 59.62% in the T4 (1200 mg/L) GO treatment. It was observed thar MDA content was enhanced in a concentration-related manner.

### Nutrient uptake

**Table 3** showed that in response to GO treatment concentrations, significant differences in the contents of nutrients were detected. The nutrient uptake increased with increasing GO concentration till T4 (1200 mg/L), while it was reduced in T5 (1500 mg/L). Moreover, the nutrient contents enhanced in a concentration-dependent manner. A significant decrease in content was found at T5 (1500 mg/L) GO, when compared to control.

**Table 3:** Effects of different GO NPs treatments on nutrient uptake of mungbean.

GO NPs treatments(mg/L)	N (mg/g)	P (mg/g)	K (mg/g)	Cu (µg/g)	Zn (µg/g)	Fe (µg/g)	Mn (µg/g)	Mo (µg/g)	B (µg/g)	Si (µg/g)
**0**	43.38 ± 0.50^c^	2.27 ± 0.02^d^	21.75 ± 1.54^bc^	51.21 ± 1.39^cd^	43.85 ± 1.03^ab^	537.33 ± 8.29^cd^	57.03 ± 1.85^bc^	2.80 ± 0.02^de^	43.13 ± 1.01^c^	260.12 ± 5.77^d^
**300**	45.73 ± 0.80^bc^	2.31 ± 0.01^cd^	22.83 ± 0.93^abc^	52.76 ± 1.11^bc^	44.36 ± 1.27^ab^	560.16 ± 11.69^bcd^	59.96 ± 1.41^abc^	2.91 ± 0.02^cd^	44.56 ± 0.64^bc^	278.63 ± 4.43^c^
**600**	47.52 ± 0.60^b^	2.36 ± 0.02^bc^	25.10 ± 0.92^ab^	53.66 ± 1.22^bc^	46.31 ± 1.17^a^	576.43 ± 7.81^abc^	61.46 ± 1.12^ab^	2.97 ± 0.03^bc^	47.63 ± 0.59^ab^	288.12 ± 3.78^bc^
**900**	50.83 ± 0.63_a_	2.42 ± 0.01^ab^	26.11 ± 0.80^ab^	55.34 ± 1.13^ab^	47.80 ± 1.15^a^	595.70 ± 8.60^ab^	62.36 ± 1.09^ab^	3.09 ± 0.04^b^	48.20 ± 0.38^a^	300.31 ± 4.56^ab^
**1200**	53.30 ± 1.18^a^	2.45 ± 0.02^a^	27.26 ± 1.01^a^	58.13 ± 1.29^a^	49.52 ± 1.35^a^	612.53 ± 10.68^a^	65.52 ± 1.12^a^	3.25 ± 0.05^a^	50.36 ± 1.25^a^	313.16 ± 5.29^a^
**1500**	38.71 ± 0.51^d^	2.34 ± 0.01^d^	18.23 ± 1.06^c^	47.69 ± 1.21^d^	39.26 ± 1.35^b^	519.58 ± 9.60^d^	53.73 ± 1.47^c^	2.71 ± 0.03^e^	38.57 ± 0.93^d^	251.33 ± 3.48^d^

Data represent the mean ± standard error. Different letters denote statistically significant differences between treatments as evaluated by the Tukey’s Multiple range test at the P<0.05 level. C= without GO NPs application (control); T1= 300 mg/L; T2= 600 mg/L; T3= 900 mg/L; T4= 1200 mg/L; T5= 1500 mg/L.

### Effect of graphene oxide nanoparticles on growth parameters of mungbean

GO NPs solution was applied to the root zone of mungbean plants over a concentration range of 300-1500 mg/L to examine the impact on plant growth in soil. Mungbean plants responded favourably to the GO NP concentrations. Their effects on different growth parameters are discussed briefly below.

### Shoot and root length

**Figure 8:**
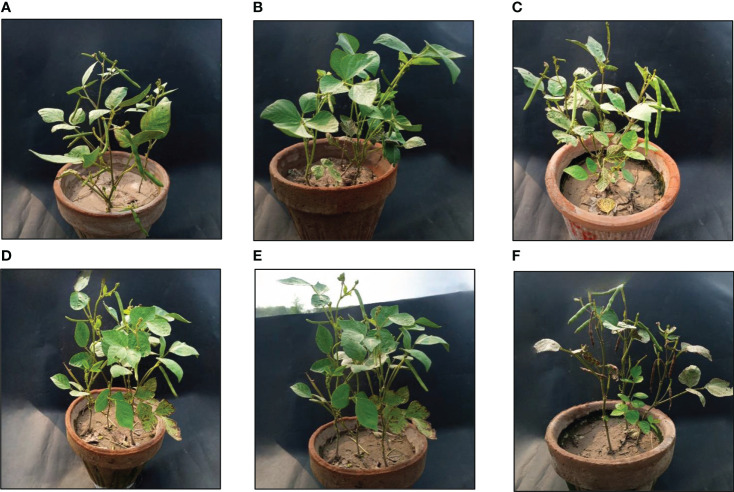
Effects of different GO NPs treatments on mungbean plants at harvesting stage. **(A)**= C= without GO NPs application (control); **(B)**= T1= 300 mg/L; **(C)**= T2= 600 mg/L; **(D)**= T3= 900 mg/L; **(E)**= T4= 1200 mg/L; **(F)**= T5= 1500 mg/L.

**Figure 9:**
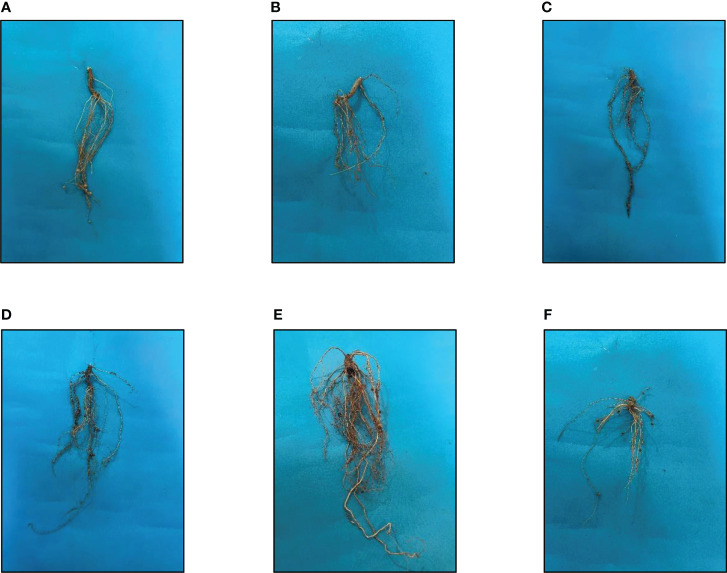
Effects of different GO NPs treatments on root lengths of mungbean. **(A)**= C= without GO NPs application (control); **(B)**= T1= 300 mg/L; **(C)**= T2= 600 mg/L; **(D)**= T3= 900 mg/L= **(E)**= T4= 1200 mg/L; **(F)**= T5= 1500 mg/L.

As a result of the application of GO NPs to mungbean, plant height showed significant differences. With the exception of doses more than 1200 mg/L, mungbean plants consistently showed greater shoot and root length than control plants (**Table 4**). When compared to the control, maximum shoot and root lengths of 38.83 cm and 18.73 cm, respectively, were recorded at 1200 mg/L. In contrast, 1500 mg/L showed the minimum shoot and root lengths of 25.83 cm and 11.1 cm respectively. Mungbean plant shoot and root images are depicted in **Figures 8**, **9**, respectively.

**Table 4:** Effect of different GO NPs treatment on growth parameters of mungbean at 75 days after sowing.

GO NPs treatments (mg/L)	Shoot length (cm)	Root length (cm)	Number of leaves (n)	Number of pods per plant (n)	No of seeds per pod (n)	Pod size (cm)	Shoot fresh weight (g)	Shoot dry weight (g)	Root fresh weight (g)	Root dry weight (g)	Root nodules (n)	Pod fresh weight	Pod dry weight
**0**	34.1 ± 0.72^b^	14.0 ± 0.72^bc^	22.0 ± 1.00^b^	6.67 ± 0.33^b^	7.67 ± 0.66^c^	6.43 ± 0.21^c^	12.9 ± 0.76^bc^	4.1 ± 0.35^b^	1.58 ± 0.06^bc^	0.48 ± 0.02^c^	10.6 ± 0.67^cd^	1.10 ± 0.04^e^	0.38 ± 0.02^cd^
**300**	35.9 ± 0.95^ab^	15.8 ± 0.95^ab^	22.6 ± 0.88^b^	7.33 ± 1.33^b^	8.67 ± 0.33^bc^	7.16 ± 0.12^bc^	13.8 ± 0.62^ab^	4.3 ± 0.24^b^	1.7 ± 0.06^b^	0.63 ± 0.02^b^	11.6 ± 0.67^bc^	1.25 ± 0.06^d^	0.42 ± 0.02^c^
**600**	37.1 ± 1.00^a^	17.0 ± 1.00^ab^	24.6 ± 0.66^ab^	8.67 ± 0.66^ab^	8.67 ± 0.33^bc^	7.76 ± 0.14^ab^	14.8 ± 0.78^ab^	4.4 ± 0.48^b^	1.8 ± 0.03^b^	0.67 ± 0.03^b^	14.0 ± 1.00^abc^	1.47 ± 0.05^c^	0.54 ± 0.03^b^
**900**	38.4 ± 1.02^a^	18.3 ± 1.02^a^	27.3 ± 0.33^a^	9.0 ± 0.57^a^	10.0 ± 0.57^ab^	7.86 ± 0.18^ab^	16.1 ± 0.66^a^	5.8 ± 0.23^a^	2.2 ± 0.08^a^	0.79 ± 0.03^a^	15.0 ± 0.57^ab^	1.67 ± 0.06^b^	0.60 ± 0.02^a^
**1200**	38.8 ± 0.83^a^	18.7 ± 0.83^a^	27.6 ± 0.66^a^	10.0 ± 0.57^a^	11.3 ± 0.33^a^	8.57 ± 0.32^a^	16.4 ± 0.66^a^	6.1 ± 0.13^a^	2.4 ± 0.05^a^	0.84 ± 0.02^a^	15.6 ± 1.20^a^	1.84 ± 0.07^a^	0.63 ± 0.04^a^
**1500**	25.8 ± 1.54^c^	11.1 ± 0.69^c^	14.0 ± 1.00^c^	5.0 ± 1.00^c^	4.67 ± 0.33^d^	4.93 ± 0.23^d^	10.1 ± 0.54^c^	3.2 ± 0.15^b^	1.2 ± 0.06^c^	0.36 ± 0.02^c^	7.33 ± 0.88^d^	1.01 ± 0.03^e^	0.33 ± 0.02^d^

Data represent the mean ± standard error. Different letters denote statistically significant differences between treatments as evaluated by the Tukey’s Multiple range test at the P<0.05 level. C= without GO NPs application (control); T1= 300 mg/L; T2= 600 mg/L; T3= 900 mg/L; T4= 1200 mg/L; T5= 1500 mg/L.

### Number of leaves

With varying GO NPs concentrations, there was a noticeable difference in the number of leaves per plant (**Table 4**). The number of leaves of the mungbean plants increased significantly when the concentrations varied from 300 to 1200 mg/L. When compared to the control, 1200 mg/L yielded the most number of leaves, while 1500 mg/L yielded the fewest.

### Number of pods per plant

The number of pods produced per plant differed statistically when compared to the control plant (**Table 4**). The application of GO NPs at 1200 mg/L resulted in an increase in the number of pods per plant by 49.92 %. While the higher concentration of 1500 mg/L resulted in decrease of number of pods by 30.13 %.

### Pod size and number of seeds per pod

**Figure 10:**
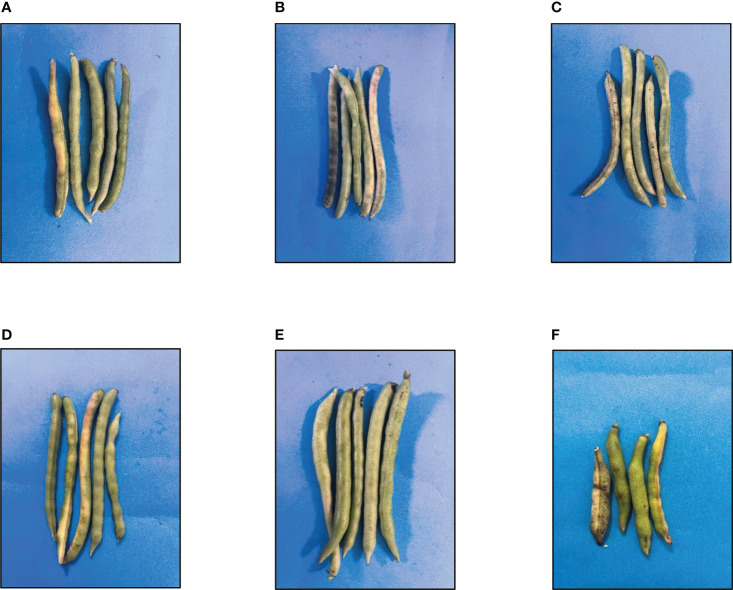
Effects of different GO NPs treatments on pod size of mungbean plants. **(A)**= C= without GO NPs application (control); **(B)**= T1= 300 mg/L; **(C)**= T2= 600 mg/L; **(D)**= T3= 900 mg/L; **(E)**= T4= 1200 mg/L; **(F)**= T5= 1500 mg/L.

The size of the pods significantly changed as the concentration of GO NPs ranged from 300 to 1500 mg/L (**Table 4**). When the concentrations increased from 300 to 1500 mg/L, the size of the pods gradually increased, and it was highest at 1200 mg/L. While it was lowest at concentrations greater than 1200 mg/L. Effect of GO NPs on pod size can be seen in **Figure 10**.

Number of seeds per pod increased significantly with increasing concentration of GO except for T5 (1500mg/L) (**Table 4**). When compared to control, the quantity of seeds per pod increased by 47.51% in T4 (1200 mg/L) but it decreased by 39.16% in T5 (1500 mg/L).

### Shoot fresh and dry weight

According to the preceding viewpoint, GO NPs applied to the soil resulted in a considerable increase in mungbean plant weights. The fresh and dry weight of shoots increased as the concentration increased over the range of 300-1200 mg/L (**Table 4**). While it decreased at a higher concentration of 1500 mg/L. Fresh and dried shoot weight increased by 27% and 49%, respectively at 1200 mg/L.

### Root fresh and dry weight

GO NPs applied to the soil resulted in a significant rise in mungbean plant root weights. When compared to the control, the maximum fresh and dry root weight was obtained at 1200 mg/L, and it increased consistently as concentrations increased from 300 to 1200 mg/L. The percentage increase in 1200mg/L concentration for root fresh and dry weight was 51.89% and 75%, respectively. While the concentration of more than 1200mg/L decreased root weight. The percentage decrease in 1500 mg/L was 18.98% for root fresh weight and 25% for root dry weight (**Table 4**).

### Root nodules

**Figure 11:**
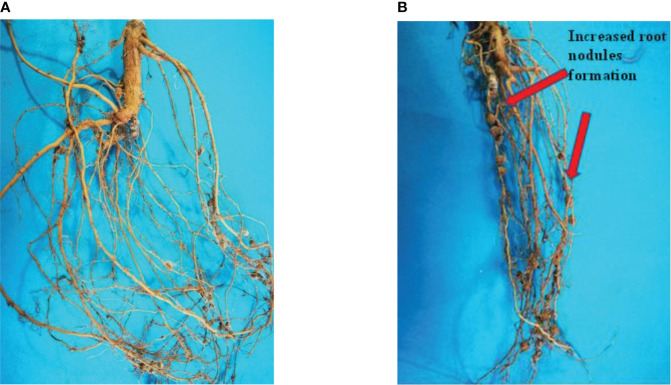
Effects of different GO NPs treatments on root nodules of mungbean plants. **(A)**= C= without GO NPs application (control); **(B)**= T4= 1200 mg/L.

Mungbean can fix atmospheric nitrogen using bacteria found in its root nodules. The rate of nodulation is quite low in most of the mungbean growing areas in Pakistan. There are several causes, but one of them appears to be poor nutrient uptake. The application of GO NPs to the soil resulted in positive root nodulation results. When compared to the control, the number of root nodules increased as the concentration increased from 300 to 1200 mg/L (**Table 4**). When compared to the control plant, the percentage increase in 1200 mg/L was 46.76%. The effect of GO NPs on root nodulation is shown in **Figure 11**.

### Pod fresh and dry weight

The application of GO NPs to the soil increased the pod weights of mungbean plants significantly. The maximum fresh and dry root weight was obtained at 1200 mg/L when compared to the control, and it rose consistently as concentrations increased from 300 to 1200 mg/L. For pod fresh and dry weight, the percentage increase in 1200mg/L concentration was 67% and 65%, respectively. While concentrations more than 1200mg/L reduced pod weight. The reduction in 1500 mg/L was 8.18 % for fresh pod weight and 13 % for dried pod weight, when compared to control (**Table 4**).

## Discussion

Understanding how nanoparticles interact with living things is crucial for biosafety due to the rapid development of nanotechnology and the production of synthetic nanomaterials. The understanding of how nanomaterials interact with plants is fundamental for assessing ecological risks, but it is also essential for the advancement of nanotechnological applications in agriculture that will increase crop yields and minimize the use of agrochemicals. Although the impacts of nano-carbon compounds on seed germination and seedling development have been widely documented, less is known about how these materials affect plant growth and life cycles of mature plants, which may differ depending on the stage of development (Nel et al., 2006). The distinct variability of nanomaterials in terms of form, size, chemical composition, solubility, aggregation, surface structure and application methods is a significant factor influencing the heterogeneity of plant responses (Petersen and Henry, 2012). The effects of graphene family nanoparticles (GFNs) on higher plants have been extensively studied, and for plants exposed to GFNs at different developmental stages, both promotion and inhibition in growth have been recorded (e.g., seed germination, root and shoot growth, and flowering). Numerous studies have shown that nanomaterials have beneficial impacts on plant development and stress tolerance when used in low doses (Mukherjee et al., 2016; Zhao et al., 2020). To determine its impact on various growth phases, we treated mungbean seedlings and matured plants with GO and it was found that exposure till 1200 mg/L GO increased plant height (root and shoot lengths), root fresh and dry weights, shoot fresh and dry weights, Number of leaves, Number of pods per plant and root nodules, but a larger dose of 1500 mg/L had a detrimental effect on seedling growth. The findings are in accordance with Song et al. (2020), which found that GN boosted seedling development at low doses but suppressed it at high doses For *Malus domestica*, 0.1 mg/L GO stimulated adventitious root development while 10 mg/L GO inhibited it (Li et al., 2018). Roots are a key organ of direct interaction between plants and soil and are crucial for plant growth due to their activities as anchors and absorbers. GO significantly influenced the development of plant roots, which may have had an impact on the growth and development of above-ground organs. According to Begum et al. (2011), graphene oxide at concentrations of 500, 1000, and 2000 mg/L was treated to the root surfaces of *Arabidopsis thaliana*, and as the concentration of graphene oxide was increased, the root length steadily declined, which ultimately effected all growth parameters. This outcome is due to the atomically thin layer of graphene oxide, which is principally a carbon-based material with a two-dimensional building block. This nanoparticle has a positive impact on plant growth (Mahmoud and Abdelhameed, 2021). Furthermore, because graphene oxide nanoparticles are smaller than cell walls, they can easily enter cell walls and perform as smart treatment-delivery systems for plant growth regulation. Graphene oxide nanomaterials influence both physiological and genetic processes in plants; as a result, it is utilized as a plant growth regulator (Chakravarty et al., 2015). Graphene oxide derivatives, like multiwalled carbon nanotubes (MWCNTs), can increase water and nutrient intake and promote plant development (Larue et al., 2012). Another factor which may have contributed to the enhanced growth in mungbean is nodules formation and development. In accordance with the previous research studies, GO NPs nanoparticles promoted root nodules and boosted the beneficial activity of soil microbiological processes (Abdallah et al., 2022). There is some evidence to support the minimal research that exists about the factors involved in the stimulatory impacts of nanoparticles on nodule formation and development. According to Burke et al. (2015), the increased nodule growth in soybean plants exposed to positively charged Fe_3_O_4_ nanoparticles was due to the availability of Fe, which is necessary for N2 fixing bacteria (Brear et al., 2013). The production of nodule factor (nodf) and genistein (a significant root-secreted isoflavone that triggers *Bradyrhizobium japonicum* nod YABC operon expression), were also found to be up-regulated by Fe_3_O_4_ NPs, which was suggested to be related to the enhanced nodulation seen in the symbiotic association between soybean plants and *Bradyrhizobium japonicum*. Photosynthesis allows plants to transform carbon dioxide into organic molecules, and pigments are required for plant growth and development (Taiz and Zeiger, 2010). A significant initial increase followed by a decrease in pigment contents was observed in mung bean treated with GO in this study which is in accordance with Siddiqui et al. (2019). The impact of GO NPs on the electron transport and energy pathways within plant cells is thought to be the cause of the increase in chlorophyll content of treated plants (Van Aken, 2015). At high GO concentrations, chlorophyll content could be reduced due to chlorophyll synthesis inhibition or chloroplast death. Because GO may penetrate algal cells and destroy organelles, it can cause damage to the chloroplast structure. (Hu et al., 2014).

MDA is a result of lipid peroxidation, and its content is related directly to ROS production (Hossain et al., 2015). MDA content showed a similar increasing tendency to antioxidant enzyme activities, implying that increased amounts of GO encouraged the formation of free radicals. The SOD, POD, and CAT activities were all well correlated with MDA content also suggesting that at higher concentration The harmful effect of GO may be caused by oxidative stress *via* ROS production. This is comparable to how graphene nanoparticles affect higher plants (Begum et al., 2011; Anjum et al., 2014). All enzyme activity and MDA levels in leaf samples consistently interacted to GO. However, other markers, such as CAT activity and MDA content, were not very sensitive at low concentrations. GO at 1200 mg/L continued to significantly boost these three enzyme activities and MDA content in leaves, with SOD, POD, CAT, and MDA levels 8.8%, 96.2%, 94.1%, and 59.6% greater than the control, respectively.

Plant growth and development are closely related to nutrient uptake efficiency. The essential macronutrients N, P, and K are the limiting components for plant growth. The micronutrients (Cu, Zn, Fe, Mn, Mo, B, and Si) are also required and engaged in a variety of metabolic pathways. In this investigation, increasing the GO dose up to 1200 mg/L resulted in an increase in the levels of N, K, Fe, Zn, Mo, B, and Si. Researchers have represented some noteworthy aspects regarding this observation. GO may inhibit nitrate uptake and buildup in plant seedling roots at higher concentrations (Weng et al., 2020) and may impact the phytoavailability of N in soils, possibly by altering soil nitrification rates and/or organic N mineralization (Duncan and Owens, 2019). As a result, it has been suggested that nanoparticles may have coated the root surface, preventing water and mineral nutrients from entering the roots and destroying the production of root hairs, inhibiting nutrient uptake at higher concentration (Hu et al., 2014; Zhao et al., 2015; Zhang et al., 2016). GO has the potential to disrupt biological metabolic systems such as amino acid, glucose, lipid, and energy metabolism, resulting in reduced nutrient absorption.

## Conclusion

Findings present in this study indicate the beneficial role of GO as plant growth promoter as well as yield enhancer within safe limits. GO formulations can be successfully incorporated in local agricuture system for better yield and profitability.

## Data availability statement

The original contributions presented in the study are included in the article/Supplementary Material. Further inquiries can be directed to the corresponding author.

## Author contributions

Conceived and designed the total experiments: Z-e-HA, TA. Performed the experiment: FM, MA, HR. Analyzed data: AA, GL. Manuscript writeup: FM. Funding procurement: GL. All authors contributed to the article and approved the submitted version.

## Funding

This study was financially supported by the Guangdong Provincial special Fund for Modern Agriculture Industry Technology Innovation Teams (Project No: 2022KJ122 and 2023KJ122) and China Young Scientist Talent Program (Project No: QN2022030024L)

## Conflict of interest

The authors declare that the research was conducted in the absence of any commercial or financial relationships that could be construed as a potential conflict of interest.

## Publisher’s note

All claims expressed in this article are solely those of the authors and do not necessarily represent those of their affiliated organizations, or those of the publisher, the editors and the reviewers. Any product that may be evaluated in this article, or claim that may be made by its manufacturer, is not guaranteed or endorsed by the publisher.
